# Gaming and Social Media Addiction in University Students: Sex Differences, Suitability of Symptoms, and Association With Psychosocial Difficulties

**DOI:** 10.3389/fpsyt.2021.740867

**Published:** 2021-10-06

**Authors:** Jonas Burén, Sissela B. Nutley, David Sandberg, Johanna Ström Wiman, Lisa B. Thorell

**Affiliations:** Department of Clinical Neuroscience, Karolinska Institutet, Stockholm, Sweden

**Keywords:** digital media addiction, internet gaming disorder, social media disorder, psychosocial difficulties, symptom severity, symptom patterns

## Abstract

**Background:** Previous research has shown that addictions to digital media can have negative impact on psychosocial health. Although Internet Gaming Disorder (IGD) has received most scholarly recognition, the potential negative consequences of Social Media Disorder (SMD) have also been found. However, few studies have assessed the symptoms of these two digital media addictions in the same way, making comparisons difficult. The present study aims to fill this gap by investigating differences and similarities regarding how common the symptoms are, sex differences, the suitability of the symptoms, and their association with psychosocial difficulties.

**Method:** A total of 688 university students (63.2% women, Mean age = 25.98) completed a questionnaire measuring symptoms of IGD and SMD, as well as psychosocial difficulties (i.e., psychosomatic symptoms, low self-concept, and social problems).

**Results:** Results showed that 1.2% of the men and 0.9% of the women met the symptom criteria for IGD (non-significant difference), whereas 3.2% men and 2.8% women met the symptom criteria for SMD (non-significant difference). Dimensional analyses indicated that men had higher IGD scores compared to women, whereas the opposite was found for SMD. Symptoms of heavy involvement in digital media (i.e., Preoccupation, Tolerance, Withdrawal, Unsuccessful attempts to control, and Escape) had high sensitivity, but low positive predictive value (PPV). However, symptoms associated with negative consequences of digital media use (i.e., Loss of interest, Continued excessive use, Deception, and Jeopardizing career/relationships) had low sensitivity, but high PPV. These symptom patterns were similar for IGD and SMD. Meeting the criteria for IGD or SMD as well as being at risk of these disorders were significantly associated with psychosocial difficulties. Symptoms of SMD generally had stronger associations with psychosomatic symptoms compared to symptoms of IGD.

**Conclusions:** We conclude that heavy involvement in digital media seems common among individuals with IGD or SMD, but also among those not meeting the symptom criteria, whereas negative consequences are less common but highly predictive of digital media addictions once present. Further attention to SMD is warranted, as it seems more common than IGD and also seems to be equally or more strongly associated with psychosocial difficulties.

## Introduction

For most individuals, using digital media is an enjoyable and stimulating activity. For some, however, digital media use can become excessive, uncontrollable, and even an addiction (1, 2). Most previous research on problematic use of digital media has focused on gaming, but there is growing evidence that social media use can also develop into an addiction (3). Recent research has demonstrated that both excessive gaming and social media use can negatively affect psychosocial health (3–5). Using a unified approach to assess gaming and social media addiction is key to distinguishing between or connecting these two digital media activities to each other as well as to investigating their association with mental health outcomes. The present study aimed to investigate and compare problematic gaming and social media use regarding how common they are, the suitability of each symptom criteria, and whether they differ in their associations with psychosocial difficulties.

### Problematic Digital Media as a Psychiatric Disorder

In the latest, fifth version of the Diagnostic and Statistical Manual of Mental Disorders (DSM-5) (6), Internet Gaming Disorder (IGD) was identified as a condition warranting further research. There are nine proposed criteria for IGD (for a full description of the criteria, see Table 1). A recent research synthesis of studies from different countries showed that around 2% of the general population may have IGD, with higher prevalence rates among younger individuals as well as among men (7). To date, social media disorder (SMD) is not officially recognized in any classification system for mental disorders.

**Table 1:** Description of each symptom for both IGD and SMD.

**Symptom**	**Symptom description according to DSM-5**	**Items included in the GSMQ^a^**
Preoccupation	The digital activity has become the dominant activity in daily life	1. I think or talk about gaming/social media even when I'm doing other things. 2. Using digital media is one of the most important activities in my life right now.
Withdrawal	Symptoms such as irritability, anxiety, or sadness when the digital activity is taken away	3. I would feel bad (irritated, anxious, sad, angry) if I was not allowed to/couldn't use digital media for a whole day.
Tolerance	The need to spend increasing amounts of time engaged with digital media	4. I need to use digital media more and more to feel satisfied.
Unsuccessful attempt to control	Inability to reduce playing, unsuccessful attempt to control, or quit the digital activity	5. I've tried to reduce the time I spend using digital media but I haven't succeeded. 6. Reducing the time I spend using digital media seems impossible for me.
Loss of interest	Loss of interest in previous hobbies and entertainment as a result of digital media	7. I skip or have quit leisure activities so I can use digital media. 8. I don't bother seeing friends so that I can use digital media instead.
Continued excessive use	Continued excessive use of the digital activity despite psychosocial problems	9. My mental health has been negatively affected by digital media (for example, sleeping problems, anger, loneliness, anxiety, unhealthy body image). 10. My use of digital media has had negative consequences, but this hasn't made me reduce it.
Deception	Deceiving family members or others about the amount of time spent on the digital activity	11. I lie about being sick so that I can stay home and use digital media. 12. I lie about how much I use digital media.
Escape	The use of digital media to relieve negative moods (e.g., helplessness, guilt, anxiety)	13. I use digital media to avoid difficult feelings. 14. I use digital media as a way to escape reality.
Jeopardizing career/relationships	Jeopardizing relationships or career/educational opportunities due to digital media use	15. My use of digital media has caused problems with my friends. 16. My use of digital media has had negative consequences for my studies/job.

a *For each item, participants are asked to provide one response for gaming and one response for social media*.

Several previous studies have demonstrated that excessive use of both gaming and social media is associated with adverse effects on individuals' psychosocial functioning and well-being. For instance, studies have linked problematic gaming to depression, anxiety, lower life satisfaction, and to negative effects on social relationships (4, 8–12). Similar links to psychosocial difficulties have also been found for problematic social media use [e.g., (5, 13, 14)]. As IGD has been identified as a psychiatric disorder, one could assume that IGD would be more strongly associated with negative psychosocial outcomes. However, to our knowledge, this issue has not been examined in previous research, at least not using similar symptom criteria for both IGD and SMD.

According to the diagnostic criteria for IGD (6), five of the nine symptom criteria should be met to be diagnosed with IGD. However, like other addictive behaviors (e.g., substance abuse and gambling), problematic gaming, and social media use most likely exist along a continuum, where the impact on psychosocial functioning becomes larger with increasing use of digital media (15). Thus, if we exclusively investigate individuals with diagnoses, only the extreme end of a continuum will be captured. This is unfortunate, as individuals with less excessive use of digital media may also experience psychosocial difficulties or be at risk of developing more severe problems later in life. It is therefore important to also identify borderline and risk groups, thereby hopefully preventing exacerbated problems.

As described above, previous research has shown that problematic use of both gaming and social media can have serious effects on mental health. However, no previous studies have included identical measures of IGD and SMD symptoms within the same study, it is still unclear whether these two types of digital media use have an equal impact on mental health. In addition, most studies investigating IGD and SMD have operationalized the two constructs differently, such as assessing SMD using fewer than nine symptoms (16). Although we agree that it is essential to keep the two constructs conceptually separate (17), we also share van den Eijnden et al.'s (3) assertion that addiction to gaming and social media are two parts of the same overarching construct and that they should therefore be defined using the same diagnostic criteria. The criteria for IGD are derived from other addictions (6), and include a generic description of symptoms that focuses on psychosocial outcomes rather than the activity per se. We therefore argue that it is sensible to employ the same approach when operationalizing SMD. Another advantage of this approach is that it enables a direct comparison between these two digital media addictions and their symptoms regarding their relation to psychosocial functioning.

### Evaluating the Importance of Different Symptom Criteria

The nine symptom criteria for IGD have been selected from the DSM-5 criteria for other addiction disorders (i.e., substance abuse or gambling). Concerns have therefore been raised regarding the extent to which all of these criteria are relevant to differentiating individuals with an actual digital addiction from individuals engaging in ordinary digital media use (18). One way of determining the suitability of each of the nine symptoms is to evaluate their sensitivity and specificity as well as their positive predictive value (PPV) and negative predictive value (NPV). Sensitivity refers to the probability of having a positive test result (in this case, a certain symptom) given that the person has the diagnosis (in this case, IGD or SMD). In contrast, specificity refers to the probability of not having a positive test result given that a person does not have the diagnosis. Positive predictive value is the probability that a person has a diagnosis given a positive test result (in this case having the symptom and also having IGD and/or SMD), and NPV is the probability that a person does not have the diagnosis given a negative test result.

Studies employing this method have typically shown that most of the nine IGD and SMD symptoms display high sensitivity among adolescents and young adults (19–21). However, high sensitivity in this context may only indicate that the symptom is common in the general population and lead to over-diagnosing. In contrast, high PPV is needed to determine whether the symptom will accurately predict whether the individual will meet the criteria for IGD or SMD. Two previous studies have shown that most of the IGD symptoms will accurately predict the disorder, with the symptoms “Unsuccessful attempts to control,” “Escape,” and “Withdrawal” having near-perfect PPV (19, 21). However, these studies employed clinical samples, which may increase the likelihood of a high PPV (22). Within a non-clinical sample, Wichstrøm et al. (23) observed considerably lower levels of sensitivity and PPV for all IGD symptoms, with “Tolerance” and “Unsuccessful attempt to control” having the lowest PPV (around 10%). The only symptoms with somewhat high PPV were “Loss of interest” and “Escape.” For SMD symptoms, PPV has, to our knowledge, not been assessed previously, but van den Eijnden et al. (3) did find acceptable sensitivity for all nine symptoms. In conclusion, only a few studies have assessed the suitability of the nine symptoms of IGD with respect to their sensitivity, specificity, and predictive power, and no previous study has directly compared IGD and SMD.

### Aims of the Present Study

The overall aim of the present study was to investigate problematic digital media use in the form of both gaming and social media use among university students, the goal being to determine how common problematic digital media use is and the suitability of each symptom criteria. Furthermore, we aimed to investigate associations between digital media use and psychosocial outcomes. More specifically, the following research questions will be addressed within the present study:

1. How common is an IGD/SMD diagnosis or IGD/SMD symptoms among university students, and are there sex differences?
2. Which symptom criteria for IGD and SMD are most indicative of an IGD or SMD diagnosis?
3. Are IGD and SMD related to psychosocial difficulties? This research question was addressed by focusing on three aspects: (1) associations between the nine different symptoms of IGD and SMD and psychosocial outcomes, (2) whether associations with psychosocial outcomes differ in strength between IGD and SMD symptoms, and (3) whether individuals meeting the full symptom criteria differ from individuals “at-risk” for IGD or SMD and individuals with few/no symptoms.

## Methods

### Participants and Procedure

The data collection occurred on two occasions during January/February 2020 and April/May 2021. On the first occasion, recruitment of university students took place at two major universities in the Stockholm area. These students received a pen-and-paper survey that they completed at the end of the class. On the second occasion, we used an online survey distributed to university students from all parts of Sweden. We contacted the students via postings in Facebook groups, face-to-face on campus areas, leaflets in student housing, and via university teachers who agreed to distribute the link to their student groups. The reason for collecting data on two different occasion was that we wanted to collect a larger, more representative sample and the first data collection had to be finished earlier than planned due to the outbreak of the COVID-19 pandemic. The two data collections generated responses from 1,040 participants, of which 191 were removed as they either only completed a minority of the questions, or did not finish the questionnaire and therefore failed to provide final consent for participation. In addition, 161 participants were removed for not meeting the inclusion criteria (i.e., not being university students). Thus, 688 participants (276 from first occasion and 412 from second occasion) who completed the questionnaire and were university students. Of these, 63.2% were women, which is a similar figure to the 61.5% share of women that are enrolled at universities in Sweden (24). The mean age for the sample was 25.98 (*SD* = 6.24). The students academic disciplines varied greatly: social sciences (27.2%), natural or medical sciences (18.6%), technology or engineering (17.3%), education (11,3%), economics (7.3%), law (6.4%), humanities (4.4%), studied other subjects or did not specify their academic disciplines (7.6%). For the full sample (i.e., including participants not gaming or using social media at all), average time per day spent gaming was 0.99 h (*SD* = 1.47) and average time per day for social media was 2.73 h (*SD* = 1.94). In addition, to assess test-retest reliability, 27.4% of the participants completed the questionnaire a second time 1–2 weeks later. No reimbursement was offered to the participants for taking part in the study.

### Measures

#### Digital Media Addiction

Digital media addiction was measured using the newly created Gaming and Social Media Questionnaire—self-rating version (GSMQ-SR). The GSMQ-SR was developed by first generating a larger number of items related to the DSM-5 criteria for IGD. In the next step, we discussed the appropriateness of these items with a smaller group of psychologists and parents. We thereafter conducted a small pilot study with 22 university students (of which five were selected because of high use of either digital games or social media) who completed a preliminary version of the GSMQ-SR and also commented on the appropriateness of each item. Finally, we selected the 16 items that we felt captured the nine symptom criteria for IGD as closely as possible (see Table 1), with two items/criteria, except for the Tolerance and Withdrawal symptoms, which were measured with one item each. The reason for only using one item each for these two criteria was that data from the pilot study showed an almost perfect overlap between the different items used to measure these two criteria. Using more than two items for each criterion was not deemed necessary and we wanted to keep the questionnaire as short as possible to allow for the questionnaire to be used within clinical settings and in combination with other rating scales. Responses to each item were made on a five-point Likert scale ranging from 0 (“Strongly disagree”) to 4 (“Strongly agree”). Data collected for 115 participants with a time interval of 1–2 weeks, showed that the test - retest reliability for both IGD and SMD symptoms was good, with Cronbach alpha values ranging from 0.76 to 0.94 and intraclass correlations (ICC) ranging from 0.77 to 0.94. The exception was Displacement for SMD, which only had moderate test-retest reliability, α = 0.58, ICC = 0.58. Participants were considered to meet a specific symptom criterion if they had a mean score (single value for Tolerance and Withdrawal) of three or higher. In line with the DSM-5 criteria for IGD (6), an IGD or SMD diagnosis required at least five of the nine symptoms. In addition, we defined an “at-risk” group as meeting three or four out of nine symptoms and a comparison group as meeting zero to two symptom criteria. In the correlation analyses, we also used the total symptom count for IGD and SMD.

#### Psychosocial Difficulties

Psychosocial difficulties were measured using three related constructs: psychosomatic problems, self-concept, and social problems. Psychosomatic problems were measured using nine items, five of which (i.e., feeling irritated/bad mood, feeling nervous/anxious, feeling down, having headaches, and having stomach aches) were selected from the HSBC symptom checklist used by WHO in well-being surveys (25). In addition, we included two items measuring sleep (“difficulty falling asleep” and “daytime tiredness”), one item measuring loneliness, and one item measuring stress. Responses to these items were made on a six-point scale with the following response alternatives: 0 = never, 1 = sometimes, 2 = once a week, 3 = twice a week, 4 = at least three times a week, 5 = every day. These nine items were then combined into a mean score. Cronbach's alpha indicated good internal reliability α = 0.89, and test-retest reliability was excellent ICC = 0.94. Self-concept (five items, e.g., “I feel bad about myself”) and social problems (six items, e.g., “I have trouble getting along with people”) were measured using the self-report version of the Weiss Functional Impairment Rating Scale (WFIRS-S). Responses to the items were made on a five-point Likert scale ranging from 0 (“not correct at all”) to 4 (“correct to a large degree”). Cronbach alpha was 0.91 for Self-concept and 0.87 for Social problems.

### Data Analysis

First, two chi-square tests were used to investigate sex differences in frequency rates for IGD and SMD. Bonferroni-corrected pairwise comparisons were performed in the event of significant group differences. Second, the suitability of each symptom of digital media use in relation to an IGD/SMD diagnosis was examined using measures of sensitivity, specificity, PPV, and NPV. Third, a series of Bonferroni-Holm corrected partial correlations with sex as a covariate was conducted to investigate the association between each symptom of IGD and SMD and psychosocial functioning. To determine whether the association with psychosocial functioning was significantly different between IGD and SMD symptoms, we used Fisher's *r* to *z* transformations with Steiger's (26) equations for asymptotic covariance of the estimate. Lastly, a series of ANCOVAs, with age and sex as covariates, was used to investigate mean differences in psychosocial functioning between the comparison group (i.e., those meeting 0–2 symptom criteria), at-risk group (i.e., those meeting 3–4 symptom criteria), and IGD/SMD group (i.e., those meeting ≥5 symptom criteria). Due to violations of normality in the dependent variables, bootstrapped (1,000 samples) standard errors and bias-corrected and accelerated (BCa) confidence intervals were created and used to interpret mean differences. Missing data were observed for age with four missing cases. Given that these four participants only represented 0.6% of the total sample, listwise deletion was used for the ANCOVAs where age was included as a covariate.

#### Controlling for Effects of COVID-19

Given that the onset of the COVID-19 pandemic occurred between the two data collection occasions, we feared this would interfere with some of the key variables in the present study (27–29). To control for this, we assessed mean differences and correlations between the two samples. We found significantly higher mean values (i.e., more difficulties) for the three measures of psychosocial difficulties for the pre-COVID sample compared to the COVID-19 sample. However, the effect sizes for these tests ranged from negligible to small (*d* range = 0.18–0.48), and no meaningful differences were found for any of the IGD and SMD variables. We also found that interrelations between the IGD/SMD symptoms and the psychosocial outcomes were not significantly different between the pre- and COVID-19 samples when comparing correlation coefficients. Thus, we concluded that the significant mean differences in the psychosocial difficulties variables would not influence the main findings of the present study, and the samples were thus pooled in subsequent analyses.

## Results

### How Common Are Symptoms of IGD and SMD?

As shown in Table 2, 1.0% of the participants belonged to the group that met the criteria (i.e., having at least five symptoms) for IGD, 2.9% for SMD, and 0.6% met the criteria for both IGD and SMD. The proportion of participants “at-risk” (i.e., having 3 or 4 symptoms) was 1.5% for IGD, 6.3% for SMD, and 0.7% were at-risk for both IGD and SMD.

**Table 2:** The proportion of men and women having each of the nine symptoms of IGD and SMD, and the proportion meeting ≥ 5 criteria, or 3 or 4 criteria.

	**Gaming**					**Social Media**				
**DSM-5 criteria**	**Total**	**Men**	**Women**	***χ^2^*** **(*df* 1)**	***V***	**Total**	**Men**	**Women**	***χ^2^*** **(*df* 1)**	***V***
	***n*** **(%)**	***n*** **(%)**	***n*** **(%)**			***n*** **(%)**	***n*** **(%)**	***n*** **(%)**		
≥5 criteria met	7 (1.0)	3 (1.2)	4 (0.9)	0.11	0.01	20 (2.9)	8 (3.2)	12 (2.8)	0.09	0.01
3 or 4 criteria met	10 (1.5)	6 (2.4)	4 (0.9)	2.36	0.06	43 (6.3)	17 (6.9)	26 (6.1)	0.16	0.02
Preoccupation	29 (4.2)	12 (4.7)	17 (3.9)	0.28	0.02	67 (9.7)	20 (7.9)	47 (10.8)	1.53	0.05
Tolerance	24 (3.5)	11 (4.3)	13 (3.0)	0.88	0.04	78 (11.3)	24 (9.5)	54 (12.4)	1.36	0.05
Withdrawal	16 (2.3)	11 (4.3)	5 (1.1)	7.20^**^	0.10	76 (11.0)	27 (10.7)	49 (11.3)	0.06	0.01
Unsuccessful attempt to control	13 (1.9)	7 (2.8)	6 (1.4)	1.67	0.05	71 (10.3)	30 (11.9)	41 (9.4)	1.02	0.04
Loss of interest	3 (0.4)	2 (0.8)	1 (0.2)	1.16	0.04	2 (0.3)	1 (0.4)	1 (0.2)	0.15	0.02
Continued excessive use	12 (1.7)	6 (2.4)	6 (1.4)	0.92	0.04	7 (1.0)	3 (1.2)	4 (0.9)	0.11	0.01
Deception	2 (0.3)	2 (0.8)	0 (0.0)	3.45^a^	0.07	77 (11.2)	27 (10.7)	50 (11.5)	0.11	0.01
Escape	75 (10.9)	35 (13.8)	40 (9.2)	3.54	0.07	75 (10.9)	29 (11.5)	66 (15.2)	1.85	0.05
Jeopardizing career/relationships	4 (0.6)	4 (1.6)	0 (0.0)	6.92^*^^a^	0.10	4 (0.6)	6 (2.4)	3 (0.7)	3.51	0.07

*χ^2^, chi-square value; df, degrees of freedom; V, Cramer's V.*

a *Fisher's Exact Test was used to calculate the p-value when expected cell count fell below 5 in at least one cell (applied on IGD Deception and IGD Jeopardizing career/relationships).*

* *p < 0.01;*

** *p < 0.001.*

As can be seen in Table 2, no significant sex differences were found with regard to the proportion meeting the cut-off for either IGD or SMD or belonging to either one of the two at-risk groups. However, as the number of men and women meeting the criteria for IGD was relatively low, we also examined sex differences for dimensional measures of IGD and SMD (i.e., mean scores on the Likert scales). The results of the *t*-tests showed that significant sex differences were found with regard to both symptoms, with men having higher scores on IGD symptoms, *t* = 5.82, *p* < 0.001, *d* = 0.51, and women had higher scores on SMD symptoms, *t* = 2.74, *p* < 0.01, *d* = 0.65.

When investigating individual symptoms, the most common symptom of IGD was Escape (10.9%), and the least common symptom was Deception (0.3%). The other symptoms of IGD ranged in frequency from 0.4 to 4.2%. Significant sex differences were found for Withdrawal and Jeopardizing career/relationships, with men being more likely than women to report these symptoms. For symptoms of SMD, Escape was once again the most commonly reported symptom (13.8%), while Loss of interest was the least commonly reported symptom (0.3%). The frequency for the other symptoms ranged from 1.0 to 11.3%, with no significant sex differences being found for any of the SMD symptoms.

### Suitability of Specific Symptoms of IGD and SMD

All participants who fulfilled the symptom criteria for IGD reported having the symptom Continued excessive use and Escape (see the “Sensitivity” column in Table 3), and a majority (57.9%) with the symptom Continued excessive use also met the cut-off for IGD (see the “PPV” column in Table 3). However, among participants with Escape symptoms, only 9.2% met the cut-off for IGD. A sizeable proportion (71.4–85.7%) of participants who met the cut-off for IGD reported having the symptoms of Tolerance, Unsuccessful attempts to control, Preoccupation, and Withdrawal, but only between 17.0 and 45.7% of those reporting these symptoms met the cut-off for IGD. For the Loss of interest symptom, the sensitivity for IGD was 42.9%, but the PPV indicated that all who met this symptom criterion met the cut-off for IGD. The sensitivity was the lowest for the Deception symptom (14.3%) and the Jeopardizing career/relationships symptom (28.6%), but nearly half of these participants met the cut-off for IGD. All values for specificity and NPV were at high and acceptable levels, which indicates that few without IGD had any of the IGD symptoms and that most individuals without IGD symptoms also did not meet the cut-off for IGD.

**Table 3:** Sensitivity, specificity, and predictive values for each IGD/SMD symptom.

	**IGD**				**SMD**			
**Symptoms**	**Sensitivity**	**Specificity**	**PPV**	**NPV**	**Sensitivity**	**Specificity**	**PPV**	**NPV**
	**(%)**	**(%)**	**(%)**	**(%)**	**(%)**	**(%)**	**(%)**	**(%)**
Preoccupation	71.4	96.5	17.0	99.7	80.0	92.4	29.3	99.2
Tolerance	85.7	97.4	24.7	99.9	85.0	91.9	29.3	99.4
Withdrawal	71.4	98.4	30.9	99.7	80.0	90.7	25.4	99.1
Unsuccessful attempt to control	85.7	99.0	45.7	99.9	90.0	91.3	29.1	99.6
Loss of interest	42.9	100	100	99.4	10.0	100	100	96.6
Continued excessive use	100	99.3	57.9	100	20.0	99.6	63.8	96.7
Deception	14.3	99.9	49.6	99.1	90.0	91.2	28.7	99.6
Escape	100	90.0	9.2	100	85.0	88.3	22.3	99.3
Jeopardizing career/relationships	28.6	99.7	49.6	99.3	25.0	99.4	62.3	97.1

A somewhat different pattern was found for SMD. A sizeable majority (range 80.0–90%) of the participants who met the cut-off for SMD met the symptom criteria for Unsuccessful attempts to control, Deception, Tolerance, Escape, Preoccupation, and Withdrawal. However, the PPV indicated that only about a fourth to a third (range 25.4–29.3%) of the participants who had these symptoms met the cut-off for SMD. A minority of the participants who met the cut-off for SMD met the symptom criteria for Loss of interest, Continued excessive use, and Jeopardizing career/relationship (range 10.0–25%), but PPV indicated that all participants with Loss of interest symptoms met the cut-off for SMD, and a large proportion with the symptoms Continued excessive use (63.8%) and Jeopardizing career/relationship (62.3%) met the cut-off for SMD. Again, specificity and NPV were at acceptable levels, albeit lower for all SMD symptoms.

### Relationships of Specific IGD and SMD Symptoms to Psychosocial Functioning

Partial correlations, controlling for sex, indicated that almost all IGD and SMD symptoms were significantly associated with all three psychosocial outcomes (see Table 4). The IGD symptoms that had the strongest associations with psychosocial difficulties were Escape (*r* = 0.29–0.41) and Continued excessive use (*r* = 0.25–0.29). However, the symptom of Withdrawal was only weakly associated with psychosomatic symptoms (*r* = 0.09) and self-concept (*r* = 0.11). Deception (*r* = 0.07) was the only symptom of IGD that was not significantly associated with social problems.

**Table 4:** Partial correlations (gender as covariate) between symptoms of IGD/SMD and psychosocial difficulties, as well as results of Fishers r to z transformations investigating differences in the strengths of the correlations.

	**Psychosomatic symptoms**			**Self-concept**			**Social problems**		
**Symptoms**	**IGD *r***	**SMD *r***	***z*** **^a^**	**IGD *r***	**SMD *r***	***z*** **^a^**	**IGD *r***	**SMD *r***	***z*** **^a^**
Preoccupation	0.14^***^	0.27^***^	−2.85^**^	0.21^***^	0.17^***^	1.02	0.17^***^	0.10^**^	1.48
Tolerance	0.17^***^	0.30^***^	−2.89^**^	0.22^***^	0.22^***^	0.05	0.21^***^	0.14^***^	1.55
Withdrawal	0.09^*^	0.22^***^	−2.74^**^	0.11^**^	0.13^***^	−0.36	0.12^**^	0.11^**^	0.13
Unsuccessful attempt to control	0.10^**^	0.29^***^	−4.27^***^	0.16^***^	0.22^***^	−1.24	0.14^***^	0.13^***^	0.24
Loss of interest	0.16^***^	0.29^***^	−3.06^**^	0.20^***^	0.20^***^	−0.17	0.23^***^	0.27^***^	−0.86
Continued excessive use	0.25^***^	0.46^***^	−5.14^***^	0.29^***^	0.39^***^	−2.43	0.26^***^	0.23^***^	0.66
Deception	0.16^***^	0.25^***^	−2.21	0.13^***^	0.20^***^	−1.79	0.07	0.10^**^	−0.77
Escape	0.35^***^	0.48^***^	−3.61^***^	0.41^***^	0.43^***^	−0.47	0.29^***^	0.31^***^	−0.46
Jeopardizing career/relationships	0.19^***^	0.28^***^	−2.10	0.24^***^	0.26^***^	−0.50	0.18^***^	0.18^***^	0.09

*r, partial correlation coefficient; z, z-value from Fisher's r to z transformation.*

a *Indicates significant differences with Holm-Bonferroni correction.*

* *p < 0.05;*

** *p <0.01;*

*** *p < 0.001.*

A similar pattern was evident for SMD symptoms, where symptoms of Escape (*r* = 0.43–0.48) and Continued excessive use (*r* = 0.39–0.46) had the strongest correlations with psychosomatic symptoms and self-concept. Symptoms of Withdrawal (*r* = 0.13–0.22) had the weakest correlations. Symptoms of Escape (*r* = 0.31) and Loss of interests (*r* = 0.27) had the strongest correlations with social problems.

When comparing the correlations between the IGD and SMD symptoms (see Table 4), the strength of the associations with psychosomatic problems was significantly higher for the SMD symptoms than for the IGD symptoms, except for the two symptoms Deception and Jeopardizing career/relationships. However, no difference in the strength of the associations between IGD and SMD symptoms was found regarding self-concept and social problems.

### Group Differences for Psychosocial Difficulties

Sex and age-adjusted mean differences between the IGD and SMD groups for each psychosocial outcome can be found in Table 5. The results from the ANCOVAs (controlling for sex and age) showed that there was a significant effect of IGD on psychosomatic symptoms, *F*_(2, 679)_ = 6.68, *p* < 001, partial η^2^ = 0.02, on self-concept, *F*_(2, 679)_ = 7.91, *p* < 0.01, partial η^2^ = 0.02, and social problems, *F*_(2, 679)_ = 8.27, *p* < 0.001, partial η^2^ = 0.02. Pairwise comparisons (see Table 6) indicated that the at-risk and the IGD groups had significantly higher mean levels of psychosocial difficulties than the comparison groups. However, no significant mean differences were found between the at-risk group and the IGD group.

**Table 5:** Adjusted means and confidence intervals for psychosocial difficulties.

	**Comparison group**		**At-risk group**		**IGD/SMD group**	
**Outcome**	**Adj. *M (SE)***	**(BCa 95% CI)**	**Adj. *M (SE)***	**(BCa 95% CI)**	**Adj. *M (SE)***	**(BCa 95% CI)**
**IGD**						
Psychosomatic symptoms	1.99 (0.04)	1.91, 2.07	2.60 (0.25)	2.06, 3.18	3.20 (0.47)	2.26, 4.22
Self-concept	2.18 (0.04)	2.10, 2.26	2.88 (0.33)	2.33, 3.48	3.45 (0.49)	2.49, 4.41
Social problems	1.66 (0.03)	1.60, 1.71	2.22 (0.24)	1.75, 2.69	2.53 (0.27)	2.02, 3.03
**SMD**						
Psychosomatic symptoms	1.92 (0.04)	1.85, 2.00	2.75 (0.15)	2.44, 3.03	3.44 (0.23)	2.93, 3.90
Self-concept	2.11 (0.04)	2.03, 2.19	2.97 (0.17)	2.63, 3.32	3.39 (0.26)	2.84, 3.91
Social problems	1.64 (0.03)	1.58, 1.69	1.97 (0.12)	1.75, 2.18	2.27 (0.21)	1.87, 2.68

*Adj. M, adjusted means; SE, standard error of adjusted mean; CI, confidence intervals*.

**Table 6:** Pairwise comparisons between the groups for psychosocial difficulties.

	**Psychosomatic symptoms**			**Self-concept**			**Social problems**		
**Outcome**	**Adj. mean difference** **(BCa 95% CI)**	***SE***	***g***	**Adj. mean difference** **(BCa 95% CI)**	***SE***	***g***	**Adj. mean difference** **(BCa 95% CI)**	***SE***	***g***
**IGD**									
At-risk group vs. Comparison	0.61^*^ (0.14, 1.06)	0.25	0.59	0.70^*^ (0.11, 1.32)	0.31	0.67	0.56^*^ (0.09, 1.03)	0.24	0.76
IGD group vs. Comparison	1.21^**^ (0.25, 2.23)	0.48	1.16	1.27^**^ (0.30, 2.24)	0.49	1.21	0.88^**^ (0.32, 1.44)	0.27	1.18
IGD group vs. At-risk group	0.60 (−0.41, 1.77)	0.54	0.61	0.57 (−0.57, 1.68)	0.58	0.51	0.32 (−0.45, 1.07)	0.35	0.43
**SMD**									
At-risk group vs. Comparison	0.83^**^ (0.53, 1.12)	0.16	0.83	0.86^**^ (0.52, 1.21)	0.17	0.86	0.33^**^ (0.08, 0.59)	0.12	0.45
SMD group vs. Comparison	1.15^**^ (1.07, 1.95)	0.24	1.52	1.28^**^ (0.80, 1.76)	0.26	1.29	0.63^**^ (0.20, 1.06)	0.21	0.86
SMD group vs. At-risk group	0.69^*^ (0.16, 1.20)	0.28	0.70	0.43 (−0.16, 1.00)	0.31	0.36	0.30 (−0.16, 0.77)	0.24	0.37

*SE, standard error of adjusted mean; CI, confidence intervals; g, Hedges' g. Comparison group for IGD n = 667, At-risk group for IGD n = 10, IGD group n = 7. Comparison group for SMD n = 622, At-risk group for SMD n = 43, SMD group n = 19.*

* *p < 0.01;*

** *p < 0.001.*

For SMD, the results of the ANCOVAs showed significant effects on psychosomatic symptoms, *F*_(2, 679)_ = 36.09, *p* < 0.001, partial η^2^ = 0.09, self-concept, *F*_(2, 679)_ = 28.31, *p* < 0.001, partial η^2^ = 0.08, and social problems *F*_(2, 679)_ = 10.67, *p* < 0.001, partial η^2^ = 0.03. Pairwise comparisons once again indicated that the at-risk and SMD group had significantly higher mean levels than the comparison group did for all outcomes. In addition, the SMD group had higher levels of psychosomatic symptoms compared to the at-risk group, but no differences between these two groups were found for self-concept or social problems.

## Discussion

The present study aimed to investigate addiction regarding gaming and social media among university students, with a special focus on comparing IGD and SMD, the suitability of individual symptom criteria, and their associations with psychosocial functioning. First, we found that a higher proportion of the participants met the criteria for SMD (2.9%) compared to IGD (1.0%), with no significant sex differences observed. Around twice as many individuals reported at-risk levels of symptoms. Second, symptoms related to heavy involvement in gaming and social media were common among participants who met the criteria for IGD and SMD (i.e., high sensitivity), but the symptoms were inaccurate in predicting IGD and SMD (i.e., low PPV). The reverse was found for symptoms related to negative consequences, with low sensitivity and high PPV. Thus, symptoms of negative consequences were less commonly reported among those with IGD/SMD but, when present, often associated with having an addiction disorder. Symptoms of Deception and Unsuccessful attempts to control were better at predicting IGD than SMD, while Preoccupation and Jeopardizing career/relationship were better at predicting SMD than IGD. Third, almost all IGD and SMD symptoms were significantly associated with our three outcome measures of psychosocial difficulties. However, symptoms of SMD had a significantly stronger association with psychosomatic symptoms compared to symptoms of IGD. Lastly, participants who met the criteria for IGD/SMD or were at-risk for IGD/SMD had more psychosocial difficulties than the comparison group, but few differences were found between the at-risk group and the group meeting the full symptom criteria.

### Symptoms of SMD Appear to Be More Common than Symptoms of IGD

Based on the operationalizations used in the present study, the likelihood of meeting the cut-off was more than three times higher for SMD than IGD. Even greater differences between IGD and SMD were found for the at-risk groups. One possible explanation for these differences in our sample is the strong dependence on the constant presence of smartphones. In contemporary society, it is practically impossible to function well-without a smartphone, and it has therefore been suggested that overcoming social media addiction can be compared to “quitting smoking while holding a lit cigarette, surrounded by smokers” [(30), p. 4]. It is also possible that the addictive features built into social media may be more effective in getting users “hooked” with their addictive features such as the use of social pressure and social comparison in apps (31), although these types of features are now becoming more and more common even in digital games.

Interestingly, we did not find any significant sex differences on the proportion of how many belonged to the IGD/SMD groups. Based on a recent meta-analysis (16), we expected that men would be more likely to meet the criteria for IGD, whereas women would be more likely to meet the criteria for SMD, albeit with small-to-medium effect sizes. In our study, it is worth noting that men had significantly higher IGD symptom scores compared to women and men were also twice as likely compared to women to be classified in the at-risk group for IGD. Thus, the lack of significant sex differences in the categorical analyses is most likely related to low power. Additionally, the meta-analysis by Su et al. (16) indicated high heterogeneity for SMD, which could be attributed to variation in how digital media use is assessed. This was not addressed in the present study, but studies have shown that men are more likely to use social media for entertainment, while women are more likely to use social media for creating social ties, communication, and information purposes [e.g., (32, 33)]. In addition, previous studies have shown that females use social media more than men (34) and that men and women use different types of social media platforms (35), as well as different type of games (36). Thus, future studies could benefit from investigating possible sex differences in *how* men and women use digital media, and *what type* of social media and games that they use. This should be considered important also in relation to psychosocial outcomes as previous research has for example shown that it is primarily passive use of social media that has negative effects [e.g., (37)] and these associations may also be moderated by sex (38, 39).

### Suitability of the Symptoms for SMD and IGD

In the present study, we investigated the suitability of the IGD and SMD symptoms by examining sensitivity, specificity, PPV, and NPV. Overall, we found high sensitivity for most of the IGD and SMD symptoms, except for the symptoms of Loss of interest, Deception and, Jeopardizing career/relationships for IGD (sensitivity below 50%). Our pattern of results is similar to that of Ko et al. (19), who found that these symptoms had the lowest sensitivity of all nine symptoms, although at higher levels than in the present study.

High sensitivity means that many individuals with the disorder report this symptom, but it can also result from the symptom being relatively common in the general population. Instead, PPV is used to indicate whether having the symptoms also predicts having IGD/SMD. In our study, PPV for the symptom Escape was only 9% for IGD and 22% for SMD. This suggests that having this symptom may not be a good indication that someone actually meets the diagnostic criteria for IGD and SMD. However, it should be noted that two previous clinical studies (19, 21) as well as a non-clinical study of children (23) have found relatively high PPV for Escape. As further discussed below, the present study also showed that Escape was strongly associated with psychosocial difficulties.

In our study, the symptom Loss of interest had the highest PPV. It has been argued that this symptom should not be regarded as a sign of addiction, because it is natural for increased interest in one activity to displace interest in other activities (40). However, similar to findings from previous studies (23, 41), Loss of Interest was not at all common (< 1%). In the present study, this symptom also had a PPV of 100% (i.e., all individuals reporting this symptom met the criteria for a diagnosis of either IGD or SMD), but low sensitivity (i.e., relatively few with the disorder reported this symptom). In conclusion, although this symptom is infrequent, Loss of interest appears to be highly predictive of IGD and SMD.

There were also a few differences between IGD and SMD regarding the suitability of specific symptoms. For the symptom Continued excessive use, the sensitivity was 100% for IGD, but only 20% for SMD. The pattern was the opposite for the symptom Deception, with high sensitivity for SMD and low sensitivity for IGD. These findings are partly in line with previous research investigating IGD (19) or SMD (3), but not with Wichstrøm et al. (23), who found a relatively low sensitivity for both symptoms. We find these differences to be somewhat unexpected, especially regarding Deception. Previous research has shown that excessive gaming can be related to social stigma (42, 43), and we would therefore expect higher levels of Deception for gaming than for social media.

In summary, our results appeared to identify two classes of addiction symptoms, which correspond to the two factors for IGD identified by Wichstrøm et al. (23). More specifically, we found high sensitivity, but low PPV, for the factor heavy involvement (i.e., Preoccupation, Tolerance, Withdrawal, Unsuccessful attempts to control and Escape), and low sensitivity, but high PPV, for the factor negative consequences (i.e., Loss of interest, Continued excessive use, Deception, and Jeopardizing career/relationships). The exceptions were Continued excessive use for IGD and Deception for SMD, which did not fit this pattern. These two classes of symptoms may indicate that symptoms suggesting overuse of gaming and social media are common, but not necessarily problematic. In contrast, symptoms suggesting real-life consequences are uncommon also among those who meet the cut-off for IGD or SMD. However, when these symptoms occur, they indicate that the individual is likely to have a more severe addiction problem.

### Association With Psychosocial Difficulties

We found that SMD symptoms, compared to IGD symptoms, generally had a stronger association with psychosomatic symptoms. These results contrast with findings from previous studies (5, 8, 44), in which only a few and negligible differences between IGD and SMD were found. The conflicting results between studies could be due to sample differences (i.e., adolescents vs. university students) and methodological differences. In our study, we employed identical measures of IGD and SMD symptoms, while previous studies have used different measures to assess IGD and SMD. Regardless of the reasons for these differences, we believe that both our own results and previous findings demonstrate that SMD symptoms should receive the same attention as IGD symptoms do, as they are both related to psychosocial difficulties.

Some of the associations found between the symptoms and psychosocial functioning deserve further consideration. First, Escape and Continued excessive use had the strongest associations with psychosocial functioning. This may be expected, because psychosocial problems are expressed within these symptoms. Second, Withdrawal was found to be only weekly associated with psychosocial functioning. This was somewhat unexpected, because this symptom is typically expressed through irritability, anxiety, and sadness. However, Withdrawal is a debated symptom of IGD. It has also been argued [e.g., (45)] that withdrawal symptoms for IGD are not equivalent to experiencing negative feelings due to restrictions imposed by external factors (i.e., a parent/partner). Rather, these studies have suggested that this symptom should reflect one's own inability to purposefully try to stop gaming. Similar to other instruments, our instrument did not take into account the reason for withdrawal symptoms. Thus, it will be important for future research to take the complexity of this symptom into account and investigate whether relations to psychosocial difficulties differ depending on how Withdrawal is operationalized.

Deception also had weak associations with psychosocial difficulties, especially social problems. Regarding IGD, this is most likely related to the fact that this symptom was very uncommon, resulting in restriction of range for this variable. It should also be noted that Deception may also depend on the individual's social relationships. Some digital media users have no one to deceive or feel no need to deceive anyone, either because their partner/friends do not care or because they can talk openly about problematic digital media use. It should also be mentioned that deception may be used to decrease conflicts, and if not caught, it may be associated with fewer social conflicts. In addition, deception may be less important for adults compared to individuals in other age groups, such as adolescents. Therefore, future studies should examine to what extent the different criteria for IGD and SMD are more or less appropriate depending on the individual's age.

### At Risk Group for IGD/SMD and Association With Psychosocial Difficulties

Both the individuals in the at-risk group and those meeting the full symptom criteria for IGD/SMD differed significantly from the comparison group regarding psychosocial difficulties. Although no significant differences were found between the at-risk and IGD/SMD groups, effect sizes were substantially larger for the IGD/SMD groups. Due to the relatively small numbers of individuals in these groups, our failure to find significant group differences is therefore likely related to low power. As previously discussed (46), prevention efforts should be initiated to identify at-risk individuals. As our study shows, these individuals have increased levels of psychosocial difficulties, and they may develop a digital media addiction later in life. It may also be important to focus on specific IGD/SMD symptoms and their differential associations with psychosocial difficulties. In the present study, we found that the symptoms Escape and Continued excessive use had up to four times higher associations with psychosocial difficulties than did other symptoms. Thus, it is possible that having a few key symptoms may be just as detrimental to the individual's health as having many of the other symptoms. Our study provides some insights into the role of different symptoms, but more studies are clearly needed.

### Limitations, Strengths, and Conclusions

Some limitations need to be acknowledged. First, although the total sample size was sufficient for most analyses, the number of individuals meeting the symptom criteria for SMD, and especially IGD, was relatively small. This may have had some impact on our results, such as creating power issues when examining sex differences and with regard to the measures of sensitivity/specificity and PPV/NPV. Therefore, our study may have failed to capture small effects, and future studies with larger samples may be needed to find these effects. Second, most participants were contacted through digital sources, possibly leading to an oversampling of avid digital media users. However, a substantial proportion of students (40.1%) were approached face-to-face, which may offset this selection bias somewhat. Although we took steps to collect data from various universities in Sweden in different academic disciplines, it is still possible that our sample was not representative of the study population, and generalizations of our results should therefore be made with caution. Third, while not the aim of the study, the cross-sectional design precludes any inference of causality. It has also been suggested [e.g., (47)] that individuals with psychosocial difficulties may be more prone to use digital media, which in turn may create reinforcing spirals that further exaggerate psychosocial difficulties. Future longitudinal studies investigating both causality and the direction of effects are welcomed. Fourth, it should be noted that the psychometric properties of the instrument used to measure IGD and SMD has not been examined with regard to its factorial and construct validity. Until future studies have validated the instrument used in this study, our results should be interpreted with caution.” However, we did show that the test-retest reliability was good for most of the symptoms. One strength with this instrument was that the items strictly followed the DSM-5 symptom criteria for IGD (6). In addition, our use of identical items to measure IGD and SMD should be considered a strength.

To conclude, the present study is, to our knowledge, the first study to directly compare IGD to SMD with regard to how common these two types of digital media addiction are, what symptoms best characterize these two types of media addiction, and how symptoms of IGD/SMD are related to psychosocial outcomes. Regarding the suitability of specific symptoms, the present study emphasizes the need to measure problematic digital media use in terms of both heavy involvement and negative consequences. Concerning relations to psychosocial outcomes, the present results indicate that a discussion is needed concerning why only IGD has been identified as a condition warranting further studies within the DSM-5 (6) when excessive social media use appears to be more common and is equally or even more strongly related to psychosocial difficulties.

## Data Availability Statement

The raw data supporting the conclusions of this article will be made available by the authors, without undue reservation.

## Ethics Statement

Ethical review and approval was not required for the study on human participants in accordance with the local legislation and institutional requirements. The participants provided written informed consent for participation in the study.

## Author Contributions

JB, LT, and SN designed the study. JB, LT, DS, and JSW collected the data. JB, LT, and DS carried out the statistical analyses. JB, LT, SN, DS, and JSW, interpreted, discussed the results, and revised the manuscript. JB and LT prepared the manuscript. All authors contributed to the article and approved the submitted version.

## Funding

The present study was supported by a Grant from the Swedish Research Council for Working Life and Welfare (Grant No. 2020-00630) to LT.

## Conflict of Interest

The authors declare that the research was conducted in the absence of any commercial or financial relationships that could be construed as a potential conflict of interest.

## Publisher's Note

All claims expressed in this article are solely those of the authors and do not necessarily represent those of their affiliated organizations, or those of the publisher, the editors and the reviewers. Any product that may be evaluated in this article, or claim that may be made by its manufacturer, is not guaranteed or endorsed by the publisher.
